# Improving disease misclassification and prevalence estimates by linking primary and secondary care electronic health records: an illustration from arthritis research

**DOI:** 10.1093/aje/kwaf206

**Published:** 2025-09-17

**Authors:** Belay Birlie Yimer, Fangyuan Zhang, Jenny Humphreys, Mark Lunt, Meghna Jani, John McBeth, William G Dixon

**Affiliations:** Faculty of Biology Medicine and Health, Centre for Epidemiology Versus Arthritis, Centre for Musculoskeletal Research, The University of Manchester, Manchester, United Kingdom; Faculty of Biology Medicine and Health, Division of Informatics, Imaging and Data Science, The University of Manchester, Manchester, United Kingdom; Faculty of Biology Medicine and Health, Centre for Epidemiology Versus Arthritis, Centre for Musculoskeletal Research, The University of Manchester, Manchester, United Kingdom; NIHR Manchester Biomedical Research Centre, Manchester University NHS Foundation Trust, Manchester Academic Health Science Centre, Manchester, United Kingdom; Faculty of Biology Medicine and Health, Centre for Epidemiology Versus Arthritis, Centre for Musculoskeletal Research, The University of Manchester, Manchester, United Kingdom; Faculty of Biology Medicine and Health, Centre for Epidemiology Versus Arthritis, Centre for Musculoskeletal Research, The University of Manchester, Manchester, United Kingdom; NIHR Manchester Biomedical Research Centre, Manchester University NHS Foundation Trust, Manchester Academic Health Science Centre, Manchester, United Kingdom; Department of Rheumatology, Salford Royal Hospital, Northern Care Alliance, Salford, United Kingdom; Faculty of Biology Medicine and Health, Centre for Epidemiology Versus Arthritis, Centre for Musculoskeletal Research, The University of Manchester, Manchester, United Kingdom; NIHR Manchester Biomedical Research Centre, Manchester University NHS Foundation Trust, Manchester Academic Health Science Centre, Manchester, United Kingdom; Faculty of Medicine, The University of Southampton, Southampton, United Kingdom; Faculty of Engineering and Physical Science, The University of Southampton, Southampton, United Kingdom; Faculty of Biology Medicine and Health, Centre for Epidemiology Versus Arthritis, Centre for Musculoskeletal Research, The University of Manchester, Manchester, United Kingdom; Faculty of Biology Medicine and Health, Division of Informatics, Imaging and Data Science, The University of Manchester, Manchester, United Kingdom; NIHR Manchester Biomedical Research Centre, Manchester University NHS Foundation Trust, Manchester Academic Health Science Centre, Manchester, United Kingdom; Department of Rheumatology, Salford Royal Hospital, Northern Care Alliance, Salford, United Kingdom

**Keywords:** psoriatic arthritis, prevalence, sensitivity, specificity, misclassification

## Abstract

Prevalence estimates using primary care data health identify cases via code lists. Validation studies can discover and exclude false positives, but it is often difficult or impossible to find false negatives. This study aimed, using the example of psoriatic arthritis (PsA), to examine the extent of and adjust for misclassification by linking primary care records with text-mined outpatient letters from a Northwest regional hospital (2014-2019). Two hundred forty-five cases of PsA were identified among 188 286 adults registered with primary care, giving an observed prevalence of 0.13% [95% CI, 0.11%-0.15%]. Among a subgroup of 7532 primary care patients attending the hospital rheumatology clinic, 202 had a primary care PsA code: 188 were confirmed as true PsA, while 14 were false positives. Primary care codes failed to identify 196 hospital-diagnosed PsA cases, leading to a more than 2-fold underestimation. The adjusted prevalence, accounting for misclassification, was 0.25% [95% CI, 0.21%-0.28%]. Linking primary care with hospital records identified false positives and negatives, enabling correction of prevalence estimates. This highlights the value of text-mining hospital letters to replace the national absence of coded secondary care diagnosis data from outpatient departments, and the importance of considering the impact of false negatives.

## Introduction

The availability of deidentified, routinely collected electronic health record (EHR) data has revolutionized medical research, allowing researchers to analyze large datasets quickly and efficiently.^1^ Primary care EHR research databanks, such as the UK Clinical Practice Research Datalink (CPRD), have been widely used for research. Obtaining valid, reliable, generalizable—and thus trustworthy—estimates from this primary care data relies heavily on accurate and complete coding of disease during usual clinical practice.

The STROBE guidelines for reporting observational research (Strengthening the Reporting of Observational Studies in Epidemiology) recommend that researchers clearly define all outcomes, exposures, predictors, potential confounders, and effect modifiers.^2^ The REporting of studies Conducted using Observational Routinely collected health Data (RECORD) statement has extended STROBE to emphasize the need for a complete list of codes and algorithms to classify exposures, outcomes, confounders, and effect modifiers from routinely collected data.^3^ Validating the accuracy of code lists and algorithms to classify variables in EHRs is crucial to avoid misclassification and inaccurate results.^1^ This should consider not only whether code lists and algorithms correctly identify cases (ie, minimizing false positives) but also whether all cases are captured (ie, minimizing false negatives).

Several methods can be used to test the validity of code lists, including internal validation (ie, by comparing overlapping data sources within a single database), statistical methods (ie, by quantifying the level of accuracy from a development study and correcting for the measured bias), and external validation.^5^^-^^7^ External validation, which involves comparing the performance of the case-finding method (eg, a codelist or more detailed algorithm of, say, clinical and medication codes) against a “gold standard”, can be done by exploring information in individual patients’ medical records. Manual case note review to understand the extent of false positives, for example by reading through hospital specialist outpatient letters, is sometimes possible but can be resource intensive. The challenge of finding false negatives*—*in other words, those patients who have the disease but lack a primary care code—is even greater. It can be likened to finding an invisible needle in a haystack. How is it possible to find someone with a particular condition if there is no recorded code for that person’s disease within their primary care EHR?

One solution is to link patient-level primary care data with secondary care data. Records from secondary care can provide more detailed information on clinical diagnoses and treatments. In the United Kingdom, however, Hospital Episode Statistics (HES) include information about diagnoses during inpatient admissions, and the occurrence of an outpatient visit—but importantly not the diagnosis at that visit. Information about diagnoses from outpatients is locked away in unstructured outpatient letters as free text and not available in any national datasets. This means accurate coded “gold standard” diagnoses for long-term conditions are absent. Recent advances in natural language processing do, however, have the potential to unlock these diagnoses and enable researchers to make use of information in outpatient letters, clinical notes, and investigations to improve the accuracy of disease classification in health data.^8^

In this paper, we explore the benefits of linking primary care and text-mined secondary care data to understand and address disease misclassification in routinely collected health data using the example of psoriatic arthritis (PsA). The aim of the analysis is to examine, then adjust for, the extent of misclassification in primary care data in Northwest England. Specific objectives are (1) to generate an estimate of the prevalence of PsA using primary care data alone, (2) in a subpopulation of patients with primary care EHR data with linked hospital rheumatology outpatient letters, to validate cases identified from primary care and quantify the number of false positives, (3) in the same subpopulation, to identify cases of PsA identified in secondary care but not coded within primary care (false negatives), and (4), in the whole primary care population, generate updated prevalence estimates to correct for the observed misclassification in the subpopulation.

## Methods

### Study setting and data sources

We used deidentified routinely collected EHR data from patients in a Northwest hospital. Primary care EHR data was available for the population of approximately 200 000 patients registered with 53 primary care practices in in a city within the Northwest region via an integrated electronic health record system. Diagnoses from hospital rheumatology specialists were available for patients who had attended the rheumatology outpatient clinic at a regional hospital, part of the Northern Care Alliance (NCA) NHS Foundation Trust.

### Study population

The primary care cohort included all adults aged ≥18 years registered to receive care from a general practice in the Northwest region, from January 1, 2014 to December 31, 2019. The secondary care cohort were all adults aged ≥18 years who attended the rheumatology outpatient clinic in this regional hospital in the same time period. Data from the hospital cohort was linked to the primary care cohort, thereby restricting to those patients who were under the care of this regional general practitioner (GP) and were seen in the local rheumatology clinic.

### Case identification

#### Primary care

Cases of PsA were identified as patients with either (1) a Read code for PsA and/or (2) meeting an algorithmic definition of probable cases without a PsA code, as described in Druce et al.^9^ This latter group included patients if they had (1) a diagnosis of psoriasis AND arthritis AND at least 1 prescription for disease-modifying anti-rheumatic drug (DMARD) treatment used for PsA, OR (2) seronegative inflammatory arthritis AND psoriasis. The detailed approach and code list is included in Table S1. Only those Read codes within the codelists and criteria above were provided to the research team, with no access to wider information on other diagnostic codes.

#### Secondary care

Outpatient letters were retrieved from the rheumatology service at a regional hospital in the Northwest. Outpatient letters are composed of 3 parts: a semistructured list of diagnosis, a semistructured list of medications, followed by an unstructured free-text “assessment” section, which were retrieved using a process as described previously.^10^ Using MedCAT software,^11^ the semistructured list of diagnoses from the outpatient letters was mapped to the Systematized Nomenclature of Medicine Clinical Terms (SNOMED CT version 2017). A SNOMED CT list presented in Table S2 was then used to identify PsA cases. The original text for all of the identified cases of PsA were then independently reviewed by clinical academic rheumatologists (W.G.D., J.H.). This confirmed the automated diagnosis by identifying and excluding any cases that included negation (eg, “no evidence of PsA”) or referred solely to family history (eg, “under investigation for knee pain: psoriasis and family history of PsA”). Case definition was agreed a priori to include cases described in the text as “probable” or “possible”. Any disagreement was resolved by a third clinical academic rheumatologist (M.J.).

### Linkage

Having identified cases in both primary care and secondary care independently as described above, the two data sources were merged, creating a dataset of patients with a GP in the Northwest region who were seen in a regional hospital rheumatology clinic between 2014 and 2019. A 2 × 2 table was generated to tabulate cases from the 2 sources. The secondary care diagnosis from the rheumatologist was considered the “gold standard”. Information for patients in the 2 discordant cells (ie, false-positive and false-negative cases) was manually reviewed to compare their primary care codes and their secondary care free-text descriptions to understand possible reasons for mismatches.

### Statistical analysis

For each data source, we identified PsA cases as the first occurrence of the relevant PsA code during the study period. Patients were assumed to be prevalent PsA cases from the first mention of diagnosis until the end of the study. The period prevalence was calculated as the number of adult (aged ≥18 years) PsA cases from January 1, 2014 to December 31, 2019, divided by the adult population (ie, the number of persons aged ≥18 years within the study window).

In the subpopulation with linked primary and secondary care diagnoses (with the secondary care diagnosis considered the “gold standard”), simple descriptive statistics were tabulated to identify the number of cases of PsA correctly identified in primary care, the number of false positives (cases identified in primary care but without a confirmatory diagnosis in the outpatient letters), and the number of false negatives (cases identified in the outpatient letters but not via the primary care codelist). Using these figures, sensitivity, specificity, positive predictive value (PPV) and negative predictive value (NPV) were calculated for primary care PsA diagnosis in this subpopulation.

Prevalence estimates for the whole of the primary care population were generated by adjusting for the observed misclassification in the subpopulation with linked data. Given a sensitivity ($\mathrm{se}$) and specificity ($\mathrm{sp}$), the observed prevalence in primary care data or expected frequency of PsA Read codes ($\mathrm{Pre}{\mathrm{v}}_{\mathrm{PC}}$) is given by:


$$ \mathrm{Pre}{\mathrm{v}}_{\mathrm{PC}}=\mathrm{se}\ast \pi +\left(1-\mathrm{sp}\right)\ast \left(1-\pi \right), $$

as where $ \pi $ is the true prevalence in the population. By rearranging this formula, we can solve for $ \pi $ as 


$$ \pi =\left(\mathrm{Pre}{\mathrm{v}}_{\mathrm{PC}}-\left(1-\mathrm{sp}\right)\right)/\left(\mathrm{se}-\left(1-\mathrm{sp}\right)\right) $$

Entering the $\mathrm{se}$ and $\mathrm{sp}$ values obtained from the subpopulation with linked primary and secondary care data and $\mathrm{Pre}{\mathrm{v}}_{\mathrm{PC}}$ from primary care data alone in the above formula, allows us to estimate the true prevalence ($\pi$) in the population. The 95% CI for the true prevalence $\left(\pi \right)$ was calculated by first determining the SE of the observed prevalence $\left(\mathrm{Pre}{\mathrm{v}}_{\mathrm{PC}}\right)$ using $\sqrt{\mathrm{Pre}{\mathrm{v}}_{\mathrm{PC}}\left(1-\mathrm{Pre}{\mathrm{v}}_{\mathrm{PC}}\right)/188286}$, followed by constructing the 95% CI for $\mathrm{Pre}{\mathrm{v}}_{\mathrm{PC}}$ with the classical CI formula $\left(\mathrm{Pre}{\mathrm{v}}_{\mathrm{PC}}\pm 1.96\mathrm{SE}\right).$ This CI was then adjusted using the above formula to account for ($\mathrm{se}$) and ($\mathrm{sp}$) of the subpopulation dataset, yielding the 95% CI for the true prevalence ($\pi$).

This approach can fail if the specificity in the linked subpopulation differs from the specificity in the total population (selection bias). If the specificity in the linked subpopulation is lower than the specificity in the total population, the expected number of false positives in the total population may exceed the observed number of positives, yielding a negative value for the number of true positives in the population and hence a negative prevalence. In this case, we have no reliable estimate of the specificity in the total population. All we know is that the total number of false positives cannot be less than the number of false positives identified in the linked subpopulation (if all unconfirmed positives are true), and cannot be greater than the number of false positives in the linked subpopulation plus the number of positives in the unlinked subpopulation (if all unconfirmed positives are false). These extreme possibilities for the number of false positives give the extreme possibilities for the specificity, which can be used in the above formula to give extremes for the true prevalence, given the observed prevalence and estimated sensitivity.

This analysis was conducted as part of the Assembling the Data Jigsaw research program. The Health Research Authority (HRA) Research Ethics Committee and Confidentiality Advisory Group granted ethical approval for the study (Reference 21/NW/0354; University of Manchester).

## Results

Primary care EHR data was available for 188 286 adults during the 6-year study window. Among the population, 52% were female. The highest proportion of patients were aged 30-49 (34%), followed by 50-64 (21%).

We identified 245 cases of PsA according to the primary care case definition. Based on a population denominator of 188 286, we estimated the observed prevalence of PsA as being 0.13% (95% CI, 0.11%-0.15%) (245/188 286).

**Figure 1:**
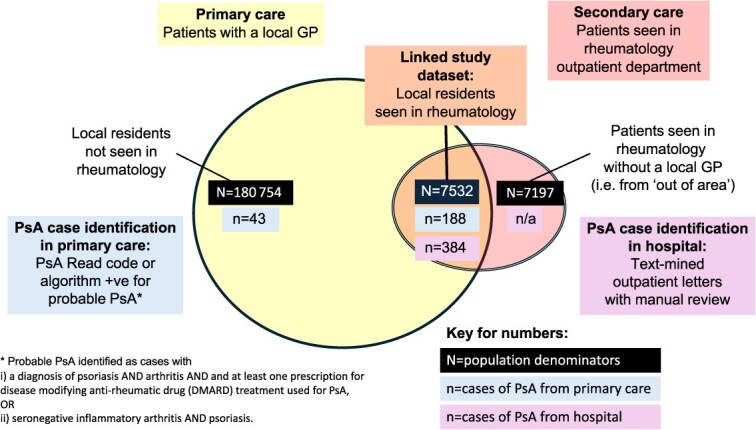
Study population and case identification in primary and secondary care.

Around 17 021 patients visited the rheumatology clinic during the 6-year period, with 590 417 outpatient letters available for a total of 14 549 patients. Linkage to primary care data was possible for 7532 of these patients—the remainder being patients seen from “out of area” (ie, who had a GP in a region outside the Northwest). Text mining of the outpatient letters identified 401 patients with a diagnostic code of PsA. Manual review of the free text for these cases led to exclusion of 17 cases, giving a total of 384 hospital cases. Cases were excluded because of negation (eg, “no evidence of PsA”) or description of a family history rather than a diagnosis of PsA. None were excluded because the algorithm had incorrectly attributed a code of PsA to an alternative diagnosis. The process of this screening and linkage is presented in Figure 1.

In the subpopulation of linked data (Table 1), there were 202 cases of PsA according to the primary care case definition. Of these, 188 cases were confirmed as having true PsA with only 14 cases being identified as false positives, that is, having a primary care code but not outpatient confirmation.

**Table 1:** Cross-classified outcomes of PSA based on primary care and secondary care diagnosis.

		**Patients seen in rheumatology outpatients**		**Patients not seen in outpatients**	**Total GP cases**	**Total**
		**Have PsA**	**No evidence of PsA**			
Patients in primary care	Code list +Ve for PsA	188	14	43	245	188 286
	Code list −Ve for PSA	196	7134	180 711	188 041	
Total		384	7148	180 754		
		7532				

We identified 196 patients who had a diagnosis in hospital but where PsA was not identified by the primary care codelist. The age and sex distribution of PsA cases identified by the hospital alone (*n* = 196) and by both the hospital and primary care (*n* = 188) were nearly identical. Therefore, of the total 384 “gold standard” cases, 45% (196/(196 + 245)) were missed using primary care codes alone. The sensitivity of the primary care codelist was 48.95% and the specificity 99.80%, with a positive predictive value of 0.93 and a negative predictive value of 0.97.

Manual review of the primary and secondary care records for the discordant cells explored reasons for mismatch, including whether the text-mining algorithm had missed cases. Reviewing the hospital free text for false positives from primary care (*n* = 14) found an absence of PsA in the majority of cases, with alternative diagnoses being described in the hospital text (eg, “psoriasis”, “psoriasis and osteoarthritis,”, or “rheumatoid arthritis” (RA) but without a diagnosis of PsA. There were a few cases (<6) where the text-mining algorithm failed to correctly pick up PsA (eg, “Peripheral spondyloarthropathy (Ps pattern)”). Unfortunately, it was not possible to review what other primary care Read codes were present, if any, in the 196 false-negative cases, as we had access only to prespecified Read codes for identification of PsA in the study dataset. There was a higher proportion of speculative diagnoses from the rheumatologists’ descriptions (ie, “possible” or “probable” cases) in the false negatives compared to the true positives, although 60% of the false negatives were cases with no diagnostic uncertainty or speculation following manual review.

Based on the formula described in the Methods section, we calculated the adjusted prevalence using the sensitivity (0.4895) and specificity (0.9980) derived from Table 1, along with the observed prevalence (0.13%). This resulted in a negative adjusted prevalence, which is not plausible. The specificity estimated from the linked data resulted in us expecting to see 362 false-positive cases in the unlinked data, whereas there were only 43 cases in total, indicating that using the specificity from the linked data directly fails to produce plausible results.^12^ However, even if we assume the worst case scenario that all 43 positives in the unmatched data are false positives, then there are 57 false positives in (180 754 + 7148) subjects without PsA, which gives a specificity of 99.97% and an adjusted prevalence of 0.21% (95% CI, 0.17%-0.24%). If none of the unverifiable positives were false positives, the specificity would be nearly 99.99%, and the adjusted prevalence would be 0.25% (95% CI, 0.21%-0.28%).

## Discussion

In this study, we explored the benefits of linking primary care and text-mined hospital outpatient data using the example of estimating the prevalence of PsA. We found only a small number of cases identified via primary care codelists that did not have the disease (false positives). Conversely, more than half of the cases identified in hospital had no primary care code (false negatives). We were unable to explore the reasons for these missed cases any further due to lack of access to the complete primary care record, although note that our initial primary care codelist and algorithm was deliberately broad to avoid missing possible cases. Our case definition in secondary care included probable and possible cases as well as certain diagnoses. The GPs may not have considered these certain enough to code as cases in their records, although this uncertainty applies to only 40% of the missed cases and therefore cannot explain much of the misclassification. Forty-three cases were identified in primary care but were never seen in the regional hospital. It is possible these patients had been under the care of hospitals in adjacent regions, had been diagnosed at our study hospital but discharged back to the GP prior to the study window, or had never been seen in hospital. It was not possible to differentiate between these options in our study.

Our prevalence estimates using primary care data alone were broadly in line with existing published literature. Studies using CPRD have estimated the annual prevalence of PsA as 0.15%-0.29% in the period 2004-2019^13^ and 0.03%-0.36% from 1991-2020.^9^ These publications provide evidence that PsA prevalence varies by calendar year, demographics, and region (eg, rates are lower in Northwest England compared to national figures^9^). This may explain some of the differences in prevalence estimates between these studies and our local sample. Furthermore, the code lists used to identify PsA differed between all three publications, which will affect case numbers. As Scott et al. note in their discussion, “our diagnosis algorithms will inevitably misclassify some people”. It is exactly this limitation that our research sought to explore.

Existing approaches to quantifying misclassification are typically limited to testing whether cases identified by applying codelists across the population truly have the disease. This is, of course, an important step. Historical studies in rheumatology have shown high proportions of false positives, for example, the study of Thomas et al. in 1987 that manually reviewed correspondence from hospitals for patients identified as having RA using a primary care diagnostic codelist.^14^ Ultimately, ~50% were found not to have proven RA. Our false-positive result of 14/7148 = 0.20% initially seems impressive, but it is higher than the observed prevalence (0.13%), so using the specificity calculated from this false-positive rate in the formula for the true prevalence gives a negative value. The true false-positive rate in the sample must therefore be lower than 0.20% as a negative value is implausible. If the true specificity is the same in the matched and unmatched samples, the specificity in the matched sample could be used as a prior in a Bayesian model to estimate the true prevalence.^15^ However, in our case, the specificity is likely to be higher in the unmatched sample than in the matched sample, since if the GP is considering the diagnosis, the patient will be referred to the hospital and hence will be in the matched sample. The model failed to converge with our data because the specificity estimated from the matched sample was totally inconsistent with the unmatched data, predicting far more false positives that the observed number of positives, and hence a negative number of true positives. We did not therefore present the results of the Bayesian model. Instead, we presented results based on the highest and lowest possible specificities compatible with our data. To obtain a valid estimate of the true prevalence, we need to consider false negatives as well as false positives—something that is usually not possible. The false-negative rate in the matched sample was 196/384 = 51%, suggesting that half of the true cases are being missed.

Other authors have explored misclassification, on occasions including the extent of false negatives. Herrett et al. reported similar findings when they identified myocardial infarctions (MIs) via 4 different data sources (primary care EHRs using CPRD, Hospital Episode Statistics (HES), the Myocardial Ischaemia National Audit Project, and national mortality data).^5^ Primary care data missed 1 in 4 nonfatal MIs, emphasizing that failure to link primary care and secondary care data can lead to biased estimates of incidence and prevalence. Although our publication explores the impact of misclassification for 1 specific disease for 1 specific purpose, namely prevalence estimates, the findings remain transferrable to using code lists to identify disease for any purpose, be it to define a study population, or to act as an exposure or an outcome.

As stated in the introduction, diagnostic information on many long-term conditions managed in outpatients are not routinely available as they are unstructured. Quantifying misclassification in our example of PsA was only possible because of the processing of free-text hospital diagnoses from outpatient letters, and linkage to the primary care data. There are several additional reasons why such analyses are not commonly conducted. Repositories of routinely collected health data often are comprised of 1 data source such as primary care electronic health records alone. Linking two routinely collected datasets for research without prior consent rightly requires a robust approval process, which in the United Kingdom is overseen by the national Confidentiality and Advisory Group^16^ with applications for linkage requiring considerable investment. Second, the process for conducting the linkage and securely storing and analyzing deidentified linked data also needs to be determined and navigated. Delivering a research project such as this thus takes substantial time, effort and resource, making it prohibitive for many researchers. Digital infrastructure programs for health data research, such as the UK Data for R&D Programme, have the potential to solve this problem by bringing multimodal data into secure data environments that follow the Five Safes principles for secure and trustworthy data access.^17^ It is promising that the recent Sudlow Review of UK health data for research^18^ recommended that a key data priority for a future national health data research service was to enhance and accelerate access to unstructured data including free text, and that the UK government has since committed to delivering a new national health data research service.^19^

Careful evaluation of the accuracy of diagnoses raises a number of challenges. The MedCAT algorithm has previously demonstrated good performance^11^ but, to avoid inaccurate automated coding, we manually reviewed all free-text descriptions of identified cases. None were falsely coded to PsA where the free text described an alternative diagnosis. We then removed any cases where the diagnoses were negated (eg, “PsA ruled out”) or related to a family history of disease. We noted that the remainder contained a spectrum of certainty in the diagnostic text, ranging from definite disease (eg, “PsA diagnosed 2016, on biologics since 2019”) to probable (eg, “treated for presumed PsA”) to possible (eg, “under investigation for possible PsA”). We chose to consider the whole spectrum from possible to definite as cases, as hospital specialists considered the diagnosis to be plausible in all such examples. Choosing instead a threshold between probable and possible is implausible given that certainty is described in various ways and terminology is used inconsistently by clinicians. Importantly, despite there being a higher proportion of possible/probable diagnoses in the false negatives compared to the true positives, definite cases still made up 60% of this group, meaning that misclassification through false negatives remains a major problem in this disease.

There are a number of implications from this research. Methods such as quantitative bias analysis are increasingly deployed in epidemiology to adjust for known limitations in the data,^20^ including misclassification. Reusing and applying prior measures of misclassification from one setting to another is a reasonable approach if assumptions hold true about the reasons for misclassification in the 2 different settings, especially given the complexity and time-consuming nature of the validation work, and that it is not always even possible in different regions. Our requirement to text mine outpatient letters shines a light on the lack of coded data about diagnoses for outpatient services in the United Kingdom. The increasing implementation of enterprise EHR systems in hospitals brings the opportunity to improve real-time clinical coding in outpatients. Lastly, the results demonstrate the value and importance of linked data across primary and secondary care. Post hoc linkage of 2 unconsented data sources for research is possible yet complex for appropriate governance reasons. The move toward shared care records offers high hopes for research, as linkage for direct care purposes removes the barrier of post hoc linkage. This linked data can then flow into secure data environments for research following deidentification.^21^

## Conclusion

The prevalence of PsA in the Northwest region was estimated as 0.13% using primary care data alone. However, in the subset of the population seen in the local rheumatology clinic, over half of the rheumatologist-diagnosed cases of PsA did not have a GP code, giving a corrected period prevalence estimate of 0.25%. This highlights the importance of careful case validation, and the benefits of doing so using linked data across primary and secondary care. Future progress in the provision of infrastructure for health data research, enabling easier access to linked data, the preprocessing of free-text information or—better still—coded data from hospitals, provides important opportunities for increasing the accuracy and the impact of population health research.

## Supplementary Material

Web_Material_kwaf206

## Data Availability

The data used in this study are subject to strict confidentiality agreements with the data provider, prohibiting public sharing. As a result, the data cannot be made available.

## Notes

Author Contributions: B.B. Yimer and F. Zhang contributed equally to this submission.
