# Supplementary material for: Improving disease misclassification and prevalence estimates by linking primary and secondary care electronic health records: an illustration from arthritis research
Source: Am J Epidemiol. 2025 Sep 17;194(12):3640–5. doi: 10.1093/aje/kwaf206 (PMC12671961; doi:10.1093/aje/kwaf206)
Supplement: Web_Material_kwaf206 [file web_material_kwaf206.docx]

**Improving disease misclassification and prevalence estimates by linking primary and secondary care electronic health records: an illustration from arthritis research**

Belay Birlie Yimer ^1*^, Fangyuan Zhang ^2*^, Jenny Humphreys ^1,4^, Mark Lunt ^1^, Meghna Jani ^1,3,4^, John McBeth ^1,4,5^, William G Dixon^1,2,3,4 †^

1. Centre for Epidemiology Versus Arthritis, Centre for Musculoskeletal Research, Faculty of Biology Medicine and Health, The University of Manchester, Manchester, UK
2. Division of Informatics, Imaging and Data Science, The University of Manchester, Manchester, UK
3. Department of Rheumatology, Salford Royal Hospital, Northern Care Alliance, Salford, United Kingdom
4. NIHR Manchester Biomedical Research Centre, Manchester University NHS Foundation Trust, Manchester Academic Health Science Centre, Manchester, UK
5. Faculty of Medicine and Faculty of Engineering and Physical Science, The University of Southampton, Southampton, UK

*These authors contributed equally to this submission.

^†^ Corresponding author.

Prof William Dixon

Vaughan House

Portsmouth Street

University of Manchester

Manchester

M13 9GB

United Kingdom

Email: [will.dixon@manchester.ac.uk](mailto:will.dixon@manchester.ac.uk)

List of included materials: Table S1 and S2

**Table S1**: the approach used to identify the psoriatic arthritis (PsA) cases from primary care dataset and the Read codelist.

| Cases of PsA in primary care dataset were identified as patients with either: | |
| --- | --- |
| 1. A Read code for PsA;   Participant has one or more single disease-specific Read code | PsA = M160 |
| And/or | |
| 1. Meeting an algorithmic definition of probable cases without a PsA code. 2. A diagnosis of psoriasis AND arthritis AND at least one prescription for disease modifying anti-rheumatic drug (DMARD) treatment used for PsA;   or   1. Seronegative inflammatory arthritis AND psoriasis | Psorasis (PsO) = M16..  Arthritis = N03…, N040.., N100.., N11F.., N06…,  DMARD_Steriods = h3…, j55.., j54.., j59.., h71.., h82.., h84.., h83.., j52.., fe7.., fe4.., j41.., j42.., j43.., j46.., fe9.., fe7.., i83.., ai3.., m40.., c67.., c64.., aa9..., c62.. |

**Table S2**: A Systematized Nomenclature of Medicine Clinical Terms (SNOMED) CT list used for the identification of psoriatic arthritis (PsA) cases from the secondary care dataset.

| A SNOMED CT list | |
| --- | --- |
| PsA cases | 156370009 |
|  | 10629311000119107 |
|  | 410482007 |
|  | 200956002 |
|  | 239812005 |
|  | 33339001 |
|  | 19514005 |
|  | 239813000 |
